# Defect-Mediated Lithium Adsorption and Diffusion on Monolayer Molybdenum Disulfide

**DOI:** 10.1038/srep18712

**Published:** 2015-12-22

**Authors:** Xiaoli Sun, Zhiguo Wang, Y. Q. Fu

**Affiliations:** 1 School of Physical Electronics, University of Electronic Science and Technology of China, Chengdu, 610054, P.R. China; 2 Department of Physics and Electrical Engineering, Faculty of Engineering and Environment, University of Northumbria, Newcastle upon Tyne, NE1 8ST, UK

## Abstract

Monolayer Molybdenum Disulfide (MoS_2_) is a promising anode material for lithium ion batteries because of its high capacities. In this work, first principle calculations based on spin density functional theory were performed to investigate adsorption and diffusion of lithium on monolayer MoS_2_ with defects, such as single- and few-atom vacancies, antisite, and grain boundary. The values of adsorption energies on the monolayer MoS_2_ with the defects were increased compared to those on the pristine MoS_2_. The presence of defects causes that the Li is strongly bound to the monolayer MoS_2_ with adsorption energies in the range between 2.81 and 3.80 eV. The donation of Li 2*s* electron to the defects causes an enhancement of adsorption of Li on the monolayer MoS_2_. At the same time, the presence of defects does not apparently affect the diffusion of Li, and the energy barriers are in the range of 0.25–0.42 eV. The presence of the defects can enhance the energy storage capacity, suggesting that the monolayer MoS_2_ with defects is a suitable anode material for the Li-ion batteries.

With increasing demand for lithium ion batteries (LIBs) to be smaller and lighter, but still to have a higher energy density, extensive research is needed to find advanced electrode materials which can provide high specific capacity, long cyclic stability, high-rate capability and safety^1^,^2^. Higher capacities of 600 to 1000  mAh/g of mono-layer graphene and their composites^3^,^4^ compared to that of the bulk counterpart of graphite (372 mAh/g)^5^ inspired researchers to search for other monolayer anode materials for the LIBs, such as graphdiyne^6^, molybdenum disulfide (MoS_2_)^7^, boron carbon nitride nanosheets^8^, and monolayer V_2_O_5_^9^, etc. MoS_2_ has a layered structure, in which the atoms are covalently bonded to form two-dimensional layers that are stacked together through weak van der Waals interactions^10^. The weak interactions between interlayers allow foreign ions or molecules to be introduced among the interlayers through intercalation without causing significant volume changes. Therefore, MoS_2_ could be developed as an intercalation host material to form a promising anode material for high energy density LIBs with a capacity of ~600 mAh/g^11^. Recently, nanostructured MoS_2_ has been attracted much attention for the anode of the LIBs, and preliminary results showed it has a higher specific capacity than that of the bulk MoS_2_^12^,^13^,^14^. The nanoflower MoS_2_ anode decorated with crumpled reduced graphene oxides exhibited a high specific capacity (1225 mAh/g) and an excellent cycling performance (680 mAh/g) after 250 cycles^13^. Ultrathin MoS_2_ nano-layers on N-doped carbon shells showed a high specific capacity of ~1000 mAh/g^14^. MoS_2_/graphene nanocomposite showed a high specific capacity of 1400 mAh/g in the first cycle and remained a specific capacity value of 1351 mAh/g after 200 cycles^12^.

Recently, two-dimensional (2D) MoS_2_ monolayer has been synthesized using different methods. It can be easily synthesized by a top-down methods by exfoliation from bulk materials, such as scotch tape based micromechanical exfoliation^15^,^16^, intercalation assisted exfoliation^17^,^18^,^19^, and liquid exfoliation^20^,^21^. The 2D MoS_2_ monolayer can also be synthesized using different bottom up approaches, such as transition metal sulfurization^22^,^23^, molybdenum oxide sulfurization^24^,^25^, physical vapor deposition^26^, and hydrothermal synthesis^27^,^28^. Defects are inevitably introduced during these fabrication processes, and could significantly affect the physical, chemical and electrical properties of the two-dimensional MoS_2_ material. The presence of defects in the monolayer MoS_2_ has been proven experimentally, and the types of defects were found to be dependent on the synthesized methods^29^,^30^,^31^,^32^. The dominant category of common defects could be changed from sulphur vacancy in the mechanical exfoliation and chemical vapor deposited samples to molybdenum antisite in the physical vapor deposited samples^30^,^32^. MoS_2_ monolayer with large areas could be synthesized using chemical vapor deposition (CVD)^29^. However, the obtained monolayer MoS_2_ is often polycrystalline in nature; thus dislocations and grain boundaries (GBs) normally appear in this monolayer^23^,^31^,^33^,^34^. The effects of intrinsic point defects (including various vacancies and antisite defects) and grain boundaries on the electronic and magnetic properties of the MoS_2_ monolayer have been investigated^34^,^35^,^36^.

The performance of the LIBs is significantly dependent on electrochemical properties of the cathode and anode materials^37^. The energy density is determined by the reversible capacity and operating voltage, which are generally determined by the electrode material chemistry, i.e., effective redox couples and maximum lithium concentration in active materials^37^. The rate capability and cycling performances are determined by the electronic and ion mobilities in the electrode materials. Apart from high energy density and fast ion mobility, electrode materials should be cheap, and also have good thermal stability which is related to the safety of LIBs^38^. Theoretical studies revealed that the Li can be stably adsorbed onto the MoS_2_ monolayer with low diffusion barriers^7^,^39^. The intrinsic point defects (including various vacancies and antisite defects) and grain boundaries appear on the MoS_2_ monolayer, however, their effects on the Li adsorption remain unexplored. In this report, for the first time as far as we know, we perform density functional theory (DFT) calculations of Li adsorption and diffusion on pristine and MoS_2_ monolayers with various intrinsic point defects and grain boundaries. To be good anode materials for the LIBs, the diffusion barriers of the Li ion in the MoS_2_ should be small, which can realize a fast charging rate. At the same time, the MoS_2_ should have a large exothermic reaction energy with the lithium so that the anode materials have a large energy storage capacity.

## Results

MoS_2_ monolayer has a sandwich-like structure with the Mo layer sandwiched between two layers of S. There are two polymorphs of MoS_2_, i.e., trigonal phase (1T) and hexagonal phase (2H). At room temperature, the MoS_2_ monolayer prefers to crystallize in a hexagonal phase^19^. The metal-stable 1T-MoS_2_ phase upon lithium and sodium intercalation has been observed by *in*-*situ* transmission electron microscopy (TEM) measurements^40^,^41^,^42^. The real mechanism of the 2H-1T phase transition is not well addressed. As discussed below, the defect formation energies in the metastable 1T-MoS_2_ have negative values, indicating that the 1T phase is not stable. Therefore, our investigations were focused mainly on the hexagonal phase structure. The calculated lattice parameters of monolayer 2H-MoS_2_ are: *a* = *b* = 3.22 Å. The Mo-S bond lengths have a constant of 2.448 Å, which is in a good agreement with experimental results^43^ and other simulation values^44^.

**Figure 1:**
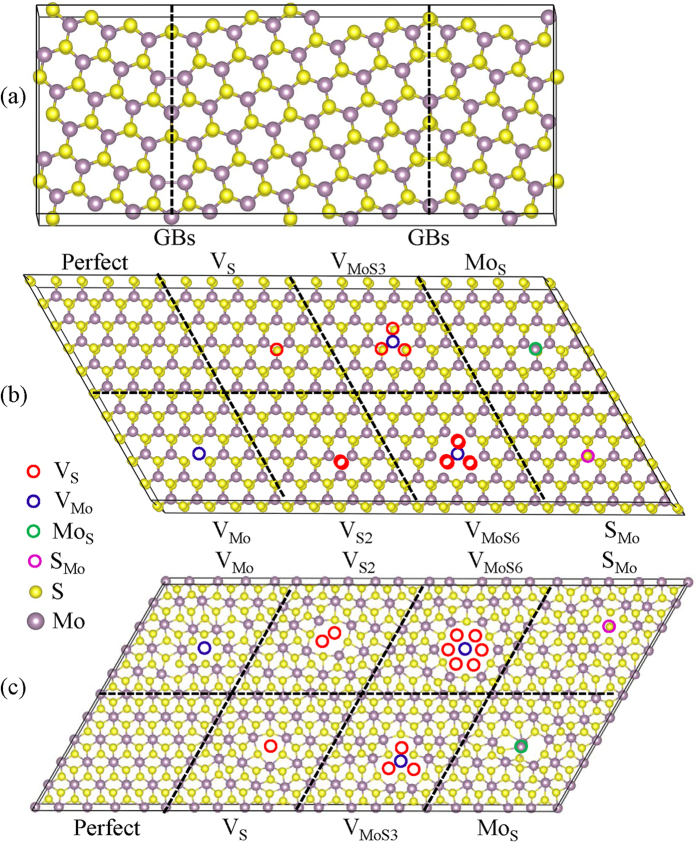
Atomistic configuration of MoS_2_ with defects. Optimized atomistic configurations of (**a**) grain boundary and defects including V_Mo_, V_S_, V_S2_, V_MoS3_, V_MoS6_, S_Mo_ in (**b**) 2H-MoS_2_ and (**c**) 1T-MoS_2_ monolayers.

We considered the commonly observed point defects in the monolayer MoS_2_^30^,^32^, including Mo (V_Mo_) and S (V_S_) single vacancies, S2 double vacancies (V_S2_), a vacancy complex of Mo and nearby three sulfur (V_MoS3_), a vacancy complex of Mo and three nearby disulfur pairs (V_MoS6_), and antisite defects where a Mo atom substituting a S atom (Mo_S_) or a S atom substituting a Mo atom (S_Mo_). The optimized defect structures from the DFT calculations in the 2H-MoS_2_ and 1T-MoS_2_ are shown in Fig. 1(b,c), respectively. The defects of atomistic structures for all the point defects including V_Mo_, V_S_, V_S2_, V_MoS3_, V_MoS6_ Mo_S_ and S_Mo_ in 2H-MoS_2_ show a 3-fold symmetry nature. Except for the point defects, an extended line defect GBs often appears in the samples prepared by a CVD method^23^,^31^,^33^,^34^. Based on direct mapping results of grain boundaries^45^,^46^ in the monolayer MoS_2_ using transmission electron microscope, the grain boundary is considered to be consisted of pentagon/heptagons (5–7) pairs with low energy configurations. The pentagon/heptagons (5–7) pairs are the basic components of GBs in the other layers materials, as evidenced by the TEM in graphene^46^,^47^. Therefore, we modeled the Li interaction with a grain boundary consisting of 5–7 polygons in the monolayers as shown in Fig. 1(a), with the GBs showing a mirror image symmetry. Each repeated grain boundary cell is composed of a Mo-rich structure with a homoelemental Mo-Mo bond, and a reversed grain boundary with a sulfur-rich structure with two S-S bonds.

### Defects in MoS_2_ monolayer

**Figure 2:**
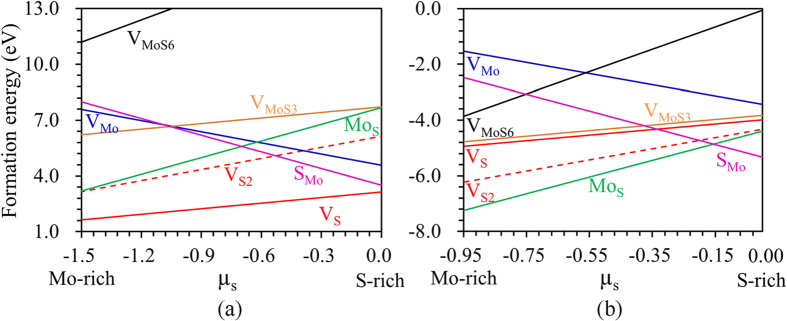
Defect formation energy. Formation energies of different point defects as a function of sulfur chemical potential for (**a**) 2H-MoS_2_ in the range −1.5 eV < *μ*_S_ < 0 eV and (**b**) 1T-MoS_2_ in the range −0.95 eV < *μ*_S_ < 0 eV, where 2H-MoS_2_ can remain stable with respect to the formation of bulk Mo (*μ*_S_ = −1.5 eV) or bulk alpha-S (*μ*_S _= 0 eV).

The chemical potentials of Mo and S in equation (1) in a thermal equilibrium are defined by: (1) the values that prevent the formation of pure elements of Mo and S, in which the chemical potential cannot be higher than the energy of bulk materials of this species; (2) a thermal equilibrium with MoS_2_ chemical potential of Mo and S, which should satisfy the equation *μ*_MoS2_ = *μ*_Mo_ + 2*μ*_S_. The defect formation energies as a function of S chemical potential, over a wide range of conditions from Mo-rich to S-rich for the 2H-MoS_2_, are shown in Fig. 2(a), where the 2H-MoS_2_ can remain stable with respect to the formation of bulk Mo (*μ*_S_ = −1.5 eV) or bulk alpha-S (*μ*_S_ = 0 eV). It can be seen that the V_S_ has the lowest formation energy, which is consistent with the experimental observation that the V_S_ is frequently observed^30^. V_S2_ is defined as a missing pair of S atoms aligned along the *c*-axis of the MoS_2_ lattice. The formation energy of the V_S2_ is about twice that of Vs, suggesting that the V_s_ does not tend to combine together. This can be supported by the fact that randomly distributed V_s_ was more frequently observed than the V_S2_^30^. The formation energy of the V_Mo_ is higher than 4.59 eV even in the S-rich condition. Under the Mo-rich condition, V_MoS3_ has a lower defect formation energy than that of the V_Mo_, which means that once the V_Mo_ is formed, the S atoms surrounding it become loose. The formation energy of large defects becomes higher as can be seen from Fig. 2(a), and the formation energy of V_MoS6_ is larger than that of V_MoS3_ for the whole chemical potentials. In the case of antisites, both the S_Mo_ and Mo_S_ have fairly low formation energy values for S-rich and Mo-rich conditions, respectively. The intrinsic defects can affect the electronic properties of the monolayer MoS_2_, thus much related work has been done to understand this effect^30^,^32^,^35^,^44^.

The defect formation energies as a function of S chemical potential over the range between Mo-rich and S-rich on 1T-MoS_2_ are shown in Fig. 2(b). All the defects have negative formation energies for the 1T-MoS_2_ in the whole S chemical potential range. The negative formation energy indicates that the defective 1T-MoS_2_ structures are more stable than the pristine ones. It is also reported that the critical value of lithium required for the stabilization of the 1T phase is estimated to x ≈ 0.4 in Li_x_MoS_2_^48^, now we will focus on how the defects affect the adsorption and diffusion the Li on the 2H-MoS_2_.

### Adsorption of Li on 2H-MoS_2_ monolayer with point defects

**Figure 3:**
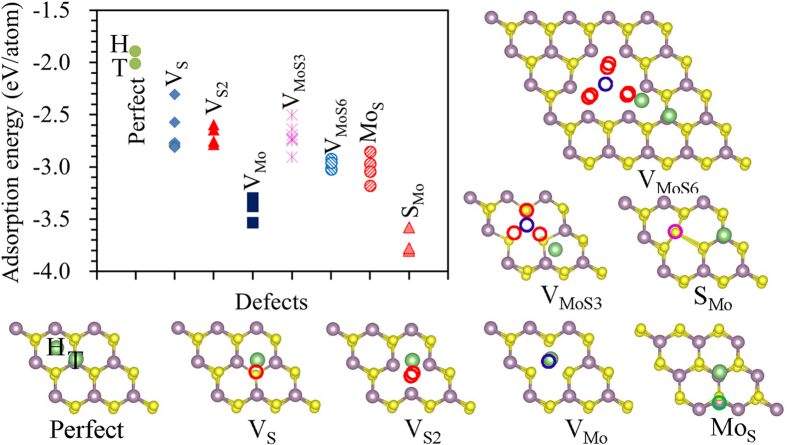
Adsorption energies and atomistic configurations. The adsorption energies of Li adsorbed on different sites around the point defect and the most stable adsorption configurations.

There are two typical adsorption positions for a Li adatom on a pristine monolayer 2H-MoS_2_: a top site above a Mo atom (T site), and the hollow site in the hexagonal center (H site), as shown in Fig. 3. The calculated adsorption energies are −2.01 and −1.90 eV for the Li at T and H sites, respectively, which showed that the Li can stay in the T site more stable than that in the H site. This agrees well with the previously reported calculation results that the T site is the most stable site for Li adsorption^39^. The calculated adsorption energy for the Li adsorption on a pristine monolayer 2H-MoS_2_ at the T site is −2.01 eV with a Li-S bond length of 2.40 Å. All the possible positions for the Li adsorption around the point defects were calculated, and the adsorption energies of the Li adsorbed onto different sites around the point defect are shown in Fig. 3, along with the lowest energy adsorption configurations. The absolute values of the adsorption energies of the Li on the defective 2H-MoS_2_ are increased compared with those on the pristine one. For example, the adsorption energy for the Li on the monolayer 2H-MoS_2_ with V_Mo_ is −3.54 eV, but this decreases to ~1.53 eV compared with the −2.01 eV for the Li adatoms on a pristine monolayer 2H-MoS_2_. The Li has favorable adsorption energy on the defective monolayer 2H-MoS_2_ than the pristine one. The strong binding energy leads to an enhanced capacity. In the presence of the V_S_ and V_S2_, the most stable site for the Li adsorption is the bridge site between two Mo atoms on the S vacancy side. The Li adatom prefers to be adsorbed on the top site above the Mo vacancy when the V_Mo_ is presented in the monolayer 2H-MoS_2_, which is similar to the site of Li adsorbed on the pristine monolayer 2H-MoS_2_, but with a Li-S bond length of 2.32 Å. The Li adatom tends to be adsorbed on the bridge site between two Mo atoms as both the V_MoS3_ and V_MoS6_ are presented in the monolayer 2H-MoS_2_. The T site is still the most stable site for the Li adsorption as both the S_Mo_ and Mo_S_ are presented in the monolayer 2H-MoS_2_.

**Figure 4:**
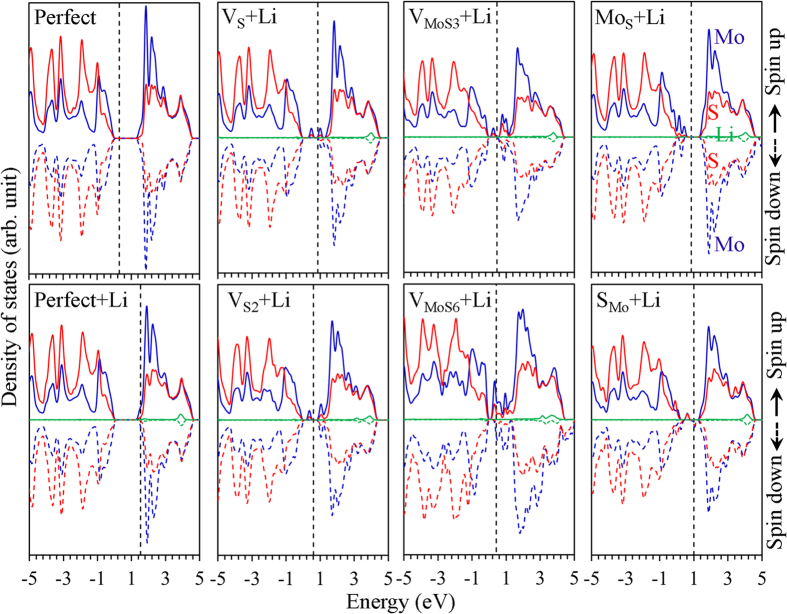
Projected density of States. Projected density of states of Li adsorbed on pristine monolayer MoS_2_ and the one with point defects.

The pristine monolayer 2H-MoS_2_ shows semiconductor properties. The valence band maximum (VBM) and conduction band minimum (CBM) are mainly composed of Mo 4*d* and S 3*p* states as seen from the projected density of states (PDOS) for both the Mo and S as shown in Fig. 4. As the Li atom is adopted on the pristine monolayer MoS_2_, the adsorption has no effect on the change of band gap and the near band edge band structure, except that the Fermi energy level moves upward into the conduction band. As the Li 2*s* state is ~3.7 eV, which means that Li is located above the CBM, the Li donates its electron to the CBM, thus pushing the Fermi level further upward. The Li adsorbed system shows an *n*-type doping state, which agrees well with the previous reports^49^. The electron transfer from the adatom to the substrate has been reported in other systems^5^,^49^,^50^,^51^. A charge transfer from K to the C layers is 0.57 electron for K adsorbed on graphene^49^. It was reported that a charge transfer of ~0.83 and 0.44 electron as Li atom adsorbed on the silicene^51^ and graphene^50^, respectively. The charge transfer will give rise to an *n*-type doping of the substrate^49^. Some peaks appear in the bandgap for the monolayer MoS_2_ with point defects, indicating that the point defects induce defect energy levels within the band gap. The Li 2*s* state locates at an energy higher than this defect levels, and the Li donates the 2*s* electron to the point defects. Therefore, the Li ion has a positive charge and the defect has a negative charge, and the adsorption was enhanced due to a strong coulomb interaction. The enhancement of Li adsorption in the defective graphene is also attributed to the same mechanism of coulomb interaction^52^.

### Adsorption of Li on 2H-MoS_2_ monolayer with grain boundaries

**Figure 5:**
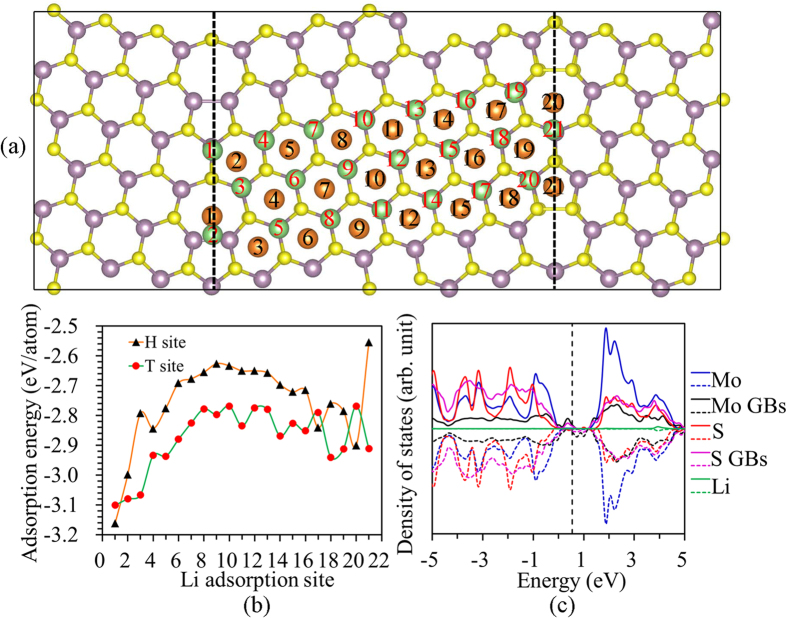
Li adsorption in MoS_2_ with GBs. (**a**) Atomistic configuration of Li atom adsorbed at different T and H sites of the monolayer MoS_2_ with grain boundaries; (**b**) Dependence of the adsorption energy of Li adatom on different sites on monolayer MoS_2_ with grain boundaries; (**c**) Site projected density of states of monolayer MoS_2_ with grain boundaries.

The calculated formation energy of grain boundary is 0.20 eV/Å. The adsorption energies for a Li atom adsorbed at the different T and H sites of the monolayer 2H-MoS_2_ with a grain boundary are shown in Fig. 5. For most of the tested sites, the Li adatom prefers to occupy the T site rather than the H site in the monolayer MoS_2_ with the grain boundary. It can be seen that a Li adatom energetically prefers the adsorption sites near the grain boundary with a Mo-Mo bond, which indicates that the grain boundaries can significantly enhance the Li atom adsorption. For example, the Li adsorption energy at the T1 site is 0.33 eV, which is lower than that at the T12 site, indicating that Li is preferred to stay at the T1 site. The site projected density of states indicates that the localized states within the band gap come from the Mo and S atoms located at the grain boundaries. The adopted Li donates its electron to the localized states, thus increasing the adsorption between the Li ion and monolayer 2H-MoS_2_.

### Diffusion of Li on 2H-MoS_2_ monolayer with defects

**Figure 6:**
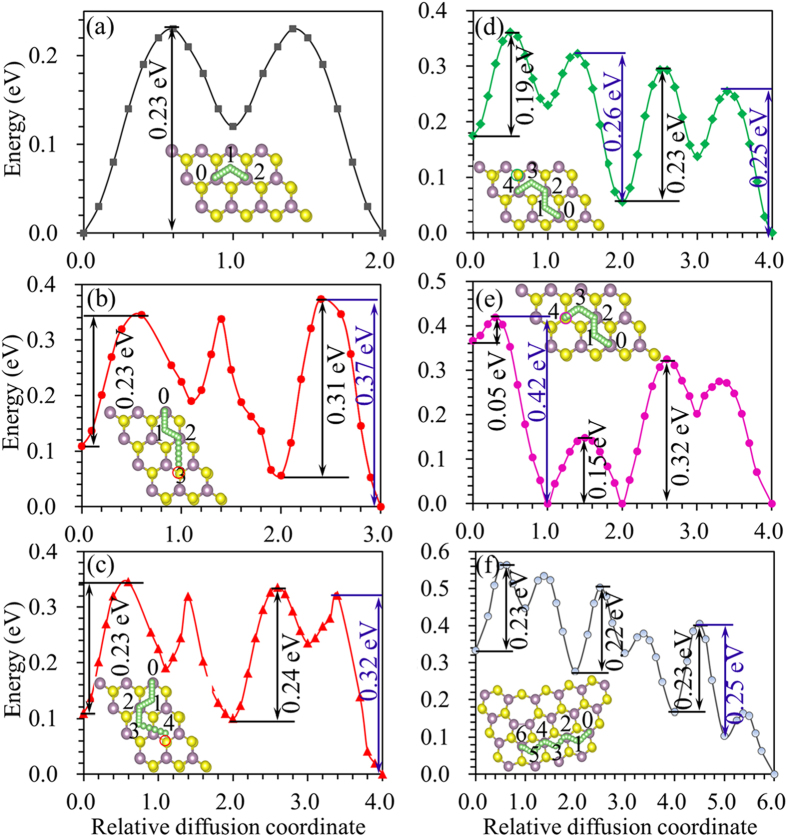
Diffusion of Li in MoS_2_ with defects. Energy curves for Li ion diffusion on (**a**) pristine 2H-MoS_2_ monolayers and the one with (**b**)/(**c**)V_S_, (**d**) Mo_S_, (**e**) S_Mo_, and (**f**) GBs defects.

As the charging rate of the LIBs relies on the Li ion mobility in the anode material, we studied the Li diffusion on the 2H-MoS_2_ monolayer with defects. As discussed above, V_S_ has the lowest formation energy in all the S chemical potential over the range between Mo-rich and S-rich, and S_Mo_ and Mo_S_ have fairly low formation energy values for S-rich and Mo-rich conditions, respectively. These defects have previously been confirmed from TEM observation^30^. We investigated the Li diffusion around the V_S_, S_Mo_, Mo_S_ and GBs defects. The nudged elastic band method (NEB)^53^ is a good choice for calculating the energy barriers, yet it needs large computation efforts. We used a constrained method to calculate the diffusion barriers. The diffusion pathway of the Li atoms was determined by moving the Li atoms along different paths with a small constant distance; and the total energy was recorded at each position. The Li atom was constrained in the direction along the path, but it is free to move in the directions perpendicular to the path, which enable the Li atom to find its optimized position. As the stable adsorption site for the Li in pristine 2H-MoS_2_ monolayer is T site, the diffusion of Li occurs from one T site to a nearest T site by passing an H site, as shown in insert of Fig. 6(a). The calculated diffusion barrier for the Li in pristine 2H-MoS_2_ monolayer is 0.23 eV, which agrees well with NEB results of 0.21^54^ and 0.24 eV^55^. Clearly, the constrained method is reliable to be used to calculate the diffusion barrier of Li in 2H-MoS_2_ monolayer. Two diffusion paths were considered for the Li diffusion in 2H-MoS_2_ monolayer with V_S_ defect, and the energy curves as a function of relative diffusion coordinates are shown in Fig. 6(b,c). The V_S_ defect does not affect the diffusion away from the defect; the energy barrier is 0.23 eV as Li diffuse from position 0 to 2. The energy barrier increases about 0.08 eV compared with the value in pristine MoS_2_ through path 1 in Fig. 6(b), and increases about 0.01 eV through path 2 in Fig. 6(c). The appearance of Mo_S_ defect does not significantly affect the diffusion behavior of Li in 2H-MoS_2_ monolayer, which can be concluded from Fig. 6(d). The S_Mo_ defect induces the increase of energy barriers up to about 0.09 eV. The energy barriers are kept at 0.23 eV as in the pristine 2H-MoS_2_ monolayer when the GBs appear. It can be seen from the energy curves in Fig. 6, the Li atom tends to diffuse to the defect position, therefore the diffusion barriers for backward diffusion of Li atoms were slightly increased. The maximum diffusion barriers are 0.37, 0.26, 0.42 and 0.30 eV for backward diffusion of Li atoms in 2H-MoS_2_ monolayer with V_S_, Mo_S_, S_Mo_ and GBs defects, respectively. From these results we can conclude that the V_S_, Mo_S_, S_Mo_ and GBs defects can enhance the adsorption of Li, but do not significantly affect the diffusion behavior of Li.

## Discussion

Defects will exist in real anode materials for LIBs, therefore, the effect of defects on the adsorption and diffusion Li in graphene^52^,^56^,^57^,^58^,^59^ and silicene^60^ has been investigated before. It was shown that the defects appear in graphene and silicene can enhance the adsorption of Li, thus can improve the Li storage capacities^56^. Together the results from this study, we can conclude that the presence of structural defects is beneficial for adsorption of Li atom in the two-dimensional materials. Li is bound to silicene with adsorption energies between 1.89 and 3.85 eV as the single vacancy, double vacancy and Stone-Thrower-Wales defects are presented^60^, which are higher than the values of 0.99–2.71 eV for the Li adopted on a defective grephene^58^. Our calculation shows that the Li can be strongly bounded to the defective 2H-MoS_2_ monolayer with adsorption energies in the range between 2.81 and 3.80 eV. The presence of the single-vacancy in graphene leads to a backward diffusion barrier of 0.56 eV^52^, and the presence of divacancy and Stone-Wales in the graphene leads to backward diffusion barriers of 0.37–0.54 eV^58^, which is higher than our calculated values of 0.26–0.42 eV for the Li in 2H-MoS_2_ monolayer with defects. The Li atom may be trapped by the defects in the graphene, thus cannot participate in the following electrochemical process. The presence of GBs in graphene leads to a decrease of about 0.92 eV in the adsorption energy of a Li adatom with diffusion barriers between 0.254 and 0.535 eV^59^. Our simulation results showed that the presence of GBs in the 2H-MoS_2_ monolayer leads a decrease of 1.2 eV in the adsorption energy of a Li adatom, and keeps the low diffusion barrier of 0.23 eV as in the pristine 2H-MoS_2_ monolayer. As the adsorption energy is higher than that in the defective graphene and the diffusion barriers are lower than that in the graphene, MoS_2_ monolayer should be a better anode material for the LIBs compared to graphene.

## Conclusion

The adsorption and diffusion of Li atom on the monolayer MoS_2_ with defects was studied using spin density functional theory. All the defects including single- and few-atom vacancies, antisite, and grain boundary can enhance the adsorption of Li atom on the monolayer MoS_2_. The donation of Li 2*s* electron to the defects causes a strong coulomb interaction, thus enhances the adsorption. High adsorption energies and small diffusion barriers for the Li in the defective MoS_2_ suggested that a monolayer MoS_2_ with defects is a suitable anode material for the Li-ion batteries.

### Simulations Details

All the calculations were performed using the spin density functional theory as implemented in the SIESTA code^61^. The electron exchange-correlation was processed using the generalized gradient approximation (GGA) with the parametrization scheme of Perdew-Burke-Ernzerhof (PBE)^62^, and the projector augmented wave (PAW) method^62^ was used to describe electron-ion interaction. Tests with a local density approximation (LDA) gave similar results for GGA in the calculations of the lattice parameters and band structures. Therefore, only the exchange-correlation potentials treated within GGA are applied for all the calculations. Electrons were described with norm-conserving Troullier-Martins pseudo-potentials^63^. The valence electron wave functions were expanded using double-ζ basis set plus polarization functions. The charge density was projected on a real space grid with a cutoff of 150 Ry to calculate the self-consistent Hamiltonian matrix elements.

A 5 × 5 hexagonal supercell of monolayer MoS_2_ was employed to model the point defects and the Li adsorption. A large spacing of 25 Å between the monolayers of MoS_2_ was used to prevent interlayer coupling. The Brillouin zone integration was modeled using a special *k*-point sampling of the Monkhorst-Pack scheme with a *Г*-centered grid. For the structural relaxation, a 3 × 3 × 1 *k*-grid was adopted for the calculations. All atomic positions and lattice constants were optimized using the conjugate gradient method until the maximum Hellmann-Feynman force acting on each atom was less than 0.02 eV/Å.

The defect formation energy *E*_*f*_ of defect *α* was calculated from the following expression^64^:


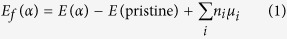


where *E*(*α*) is the total energy of the supercell containing a relaxed defect (vacancy, vacancy complex and antisite), *E*(pristine) is the total energy of the same supercell without defects, *μ*_*i*_ is the chemical potential of species *i*. *n*_*i*_ is the number of exchanged particles between the supercell and the reservoirs in forming the defect cell. The formation energy of grain boundary was calculated using *E*_*f*_ = (*E*_grainbounary_−*E*_pristine_)/*L*_*GB*_, where *E*_grainboundary_ and *E*_pristine_ are the total energy values of a pristine MoS_2_ monolayer and the one with the grain boundary having the same number of MoS_2_ atoms pairs, and *L*_*GB*_ is the length of the grain boundary.

To analyze the stability of Li adsorbed on MoS_2_ with point defects, the adsorption energy of a Li adatom was calculated using


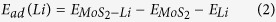


where 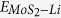 and 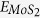
are the total energies of MoS_2_ with and without Li-adsorption, respectively. 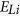 is the energy of an isolated lithium atom. According to the definition, a more negative binding energy indicates a more favorable exothermic reaction between the monolayer MoS_2_ and Li.

## Additional Information

**How to cite this article**: Sun, X. *et al.* Defect-Mediated Lithium Adsorption and Diffusion on Monolayer Molybdenum Disulfide. *Sci. Rep.* **5**, 18712; doi: 10.1038/srep18712 (2015).
